# Supplementary material for: Impact of Institutional Monthly Volume of Transcatheter Edge-to-Edge Repair Procedures for Significant Mitral Regurgitation: Evidence from the GIOTTO-VAT Study
Source: Medicina (Kaunas). 2025 May 16;61(5):904. doi: 10.3390/medicina61050904 (PMC12112984; doi:10.3390/medicina61050904)
Supplement: Supplementary file 1 [file medicina-61-00904-s001.zip › medicina-3578191-supplementary.pdf]

Supplementary Materials

Table S1. Key outcomes according to tertiles of total volume, center-wise.

Outcome	First tertile	Second tertile	Third tertile	P
Patients	674	709	830	-
Cases per center	55 (45;69)	127 (123; 160)	281 (245; 311)	-
Device success	657 (97.5%)	701 (98.9%)	803 (96.8%)	0.017
Procedural success	628 (93.2%)	691 (97.5%)	800 (96.4%)	<0.001
Inhospital death	13 (1.9%)	19 (2.7%)	30 (3.6%)	0.149
Inhospital bleeding	2 (0.3%)	7 (1.0%)	11 (1.3%)	0.018
Inhospital vascular complication	3 (0.5%)	3 (0.4%)	10 (1.2%)	0.148
Days of hospitalization	4 (3; 5)	4 (3; 6)	7 (6; 10)	<0.001
Follow-up outcomes				
Death	163 (24.2%)	179 (25.3%)	197 (23.7%)	0.783
Cardiac death	88 (13.1%)	96 (13.5%)	102 (12.3%)	0.758
Death or hospitalization for heart failure	219 (32.5%)	213 (30.0%)	253 (30.5%)	0.575
Cardiac death or hospitalization for heart failure	131 (19.4%)	96 (13.5%)	132 (15.9%)	0.012

Table S2. Key outcomes according to the first 50 cases versus the subsequent ones, center-wise.

Outcome	First 50 cases	Subsequent ones	P
Patients	937	1276	-
Device success	917 (97.9%)	1244 (97.5%)	0.670
Procedural success	885 (94.5%)	1234 (96.7%)	0.010
Inhospital death	26 (2.8%)	36 (2.8%)	1.0
Inhospital bleeding	7 (0.7%)	13 (1.0%)	0.084
Inhospital vascular complication	6 (0.6%)	10 (0.8%)	0.803
Days of hospitalization	6 (5; 10)	5 (4; 7)	<0.001
Follow-up outcomes			
Death	310 (33.1%)	229 (18.0%)	<0.001
Cardiac death	156 (16.7%)	130 (10.2%)	<0.001
Death or hospitalization for heart failure	385 (41.1%)	300 (23.5%)	<0.001
Cardiac death or hospitalization for heart failure	191 (20.4%)	168 (13.2%)	<0.001

Table S3. Unadjusted and adjusted analysis according to tertiles of total volume, center-wise.*.

Outcome	Unadjusted effect estimates	Adjusted effect estimates
Death		
Tertile 2 vs 1	0.76 (0.61-0.94), p=0.011	0.86 (0.58-1.27), p=0.440
Tertile 3 vs 1	0.94 (0.76-1.16), p=0.533	0.76 (0.53-1.10), p=0.144
Tertile 3 vs 2	1.24 (1.01-1.52), p=0.038	0.89 (0.61-1.32), p=0.570
Cardiac death		
Tertile 2 vs 1	0.77 (0.57-1.04), p=0.083	0.82 (0.49-1.38), p=0.462
Tertile 3 vs 1	0.91 (0.68-1.21), p=0.517	0.68 (0.42-1.10), p=0.114
Tertile 3 vs 2	1.18 (0.89-1.57), p=0.251	0.82 (0.49-1.40), p=0.474

Death or hospitalization for heart failure		
Tertile 2 vs 1	0.73 (0.60-0.88), p=0.001	0.67 (0.46-0.96), p=0.030
Tertile 3 vs 1	0.92 (0.77-1.10), p=0.361	0.72 (0.52-1.00), p=0.048
Tertile 3 vs 2	1.27 (1.05-1.52), p=0.012	1.08 (0.74-1.57), p=0.688
Cardiac death or hospitalization for heart failure		
Tertile 2 vs 1	0.57 (0.44-0.75), p<0.001	0.47 (0.28-0.79), p=0.004
Tertile 3 vs 1	0.82 (0.64-1.04), p=0.108	0.69 (0.45-1.06), p=0.089
Tertile 3 vs 2	1.42 (1.09-1.86), p=0.009	1.46 (0.86-2.48), p=0.162

*reported as hazard ratio (95% confidence interval), p value.

Table S4. Unadjusted and adjusted analysis according to the first 50 cases versus the subsequent ones, center-wise (hazard ratios <1 indicate better outcomes after the first 50 cases).*.

Outcome	Unadjusted effect estimates	Adjusted effect estimates
Death	0.80 (0.67-0.95), p=0.010	1.67 (0.78-3.57), p=0.187
Cardiac death	0.85 (0.67-1.08), p=0.194	2.62 (0.85-8.09), p=0.093
Death or hospitalization for heart failure	0.75 (0.64-0.87), p<0.001	1.13 (0.59-2.20), p=0.709
Cardiac death or hospitalization for heart failure	0.76 (0.62-0.95), p=0.013	1.77 (0.66-4.76), p=0.257

*reported as hazard ratio (95% confidence interval), p value.

Table S5. Cut-offs for institutional volume of transcatheter edge-to-edge repair (TEER) proposed or analyzed in the scholarly literature.

PMID	Cut-off	Year	Evidence/recommendation source	Limitations
33903039	None evident	2017	US Nationwide Readmissions Database	Limited to hospital outcomes
31320029	>50 cases (with improvements up to 200)	2019	US TVT Registry	Ample variability in volume
31857196	>20 TEER per year, >40 TEER in the prior 2 years	2019	US AATS/ACC/SCAI/STS guideline	Limited evidence based
37499594	None evident	2019	US Nationwide Readmissions Database	Limited to 1-month outcomes
37085122	>8 cases per year (possibly 24)	2019	US Nationwide Readmissions Database	Limited to 6-month outcomes
33759319	None evident	2021	German TEER registry	Limited to hospital outcomes
